# Targeting endocytosis to sensitize cancer cells to programmed cell death

**DOI:** 10.1042/BST20231332

**Published:** 2024-08-02

**Authors:** Emily T. Chan, Cömert Kural

**Affiliations:** 1 Interdisciplinary Biophysics Graduate Program, The Ohio State University, Columbus, OH 43210, U.S.A; 2 Department of Physics, The Ohio State University, Columbus, OH 43210, U.S.A

**Keywords:** cancer, cell death, endocytosis, immune evasion, membrane mechanics

## Abstract

Evading programmed cell death (PCD) is a hallmark of cancer that allows tumor cells to survive and proliferate unchecked. Endocytosis, the process by which cells internalize extracellular materials, has emerged as a key regulator of cell death pathways in cancer. Many tumor types exhibit dysregulated endocytic dynamics that fuel their metabolic demands, promote resistance to cytotoxic therapies, and facilitate immune evasion. This review examines the roles of endocytosis in apoptotic resistance and immune escape mechanisms utilized by cancer cells. We highlight how inhibiting endocytosis can sensitize malignant cells to therapeutic agents and restore susceptibility to PCD. Strategies to modulate endocytosis for enhanced cancer treatment are discussed, including targeting endocytic regulatory proteins, altering membrane biophysical properties, and inhibiting Rho-associated kinases. While promising, challenges remain regarding the specificity and selectivity of endocytosis-targeting agents. Nonetheless, harnessing endocytic pathways represents an attractive approach to overcome apoptotic resistance and could yield more effective therapies by rendering cancer cells vulnerable to PCD. Understanding the interplay between endocytosis and PCD regulation is crucial for developing novel anticancer strategies that selectively induce tumor cell death.

## Introduction

Cancer remains a leading cause of mortality worldwide, with many tumor types exhibiting resistance to standard chemotherapies and radiation treatments that aim to induce cancer cell death [1,2]. Overcoming this therapeutic challenge requires developing novel strategies that not only induce or enhance cancer cell death but also circumvent mechanisms of resistance inherent in conventional treatments [3–5]. Unlike traditional approaches that broadly target cellular proliferation, these new strategies focus on selectively exploiting vulnerabilities within cancer cells to initiate programmed cell death (PCD), while preserving healthy tissues.

Endocytosis, the mechanism by which cells internalize extracellular materials and molecules, has emerged as an attractive target for cancer therapy [6–9]. Cancer cells often exhibit dysregulated endocytic pathways that support their increased metabolic demands and rapid division [6,10]. For example, many cancer cells overexpress receptors like the transferrin receptor or growth factor receptors, which are internalized via clathrin-mediated endocytosis (CME) to fuel tumor growth [7,9,11]. Disrupting endocytosis in these malignant cells can deprive them of essential nutrients and signaling factors, thereby sensitizing them to PCD [12]. Furthermore, inhibiting endocytosis can prevent the internalization and trafficking of therapeutic agents, rendering cancer cells more susceptible to cytotoxic drugs and other anti-cancer modalities [9,13].

In this review, we first define PCD and the mechanisms of how cancer cells evade this process. We will then examine the current understanding of how endocytic pathways are altered in cancer and discuss strategies to target these processes as a means to enhance cancer cell susceptibility to PCD. We highlight recent studies demonstrating the therapeutic potential of modulating endocytosis in various tumor types. Finally, we consider the challenges and future directions in translating approaches targeted at endocytic pathways into effective cancer treatments that selectively trigger cell death in tumors.

## PCD in cancer

Cancer is a complex and heterogeneous disease that is characterized by uncontrolled cell proliferation and evasion of cell death mechanisms [14–17]. PCD, composed of apoptosis, autophagy, and programmed necrosis, is essential for maintaining tissue homeostasis and eliminating aberrant cells [5,17]. However, in cancer, the balance between cell survival and death is disrupted, leading to tumor progression and treatment resistance [3,16,17].

Apoptosis, the most extensively studied form of PCD, serves as a critical mechanism for eliminating damaged or unwanted cells. The process of apoptosis is orchestrated by two primary pathways: the extrinsic pathway and the intrinsic pathway [18–20]. In the extrinsic pathway, external death signals activate death receptors on the cell surface, such as Fas (CD95) and tumor necrosis factor receptor 1, leading to the formation of the death-inducing signaling complex (DISC) [21–23]. The DISC recruits and activates procaspase-8, initiating a cascade of caspase activation and ultimately resulting in cell death. Conversely, the intrinsic pathway is initiated by intracellular stress signals, such as DNA damage or metabolic imbalance, leading to mitochondrial outer membrane permeabilization [20,24]. This process releases cytochrome c into the cytosol, activating the apoptosome and triggering caspase activation. Dysregulation of apoptotic pathways in cancer often occurs through genetic mutations, epigenetic alterations, or dysregulated expression of apoptosis-related proteins, allowing cancer cells to evade apoptotic signals and promote tumor survival [16,17].

Autophagy is a conserved catabolic process and plays a dual role in cancer biology. Under physiological conditions, autophagy maintains cellular homeostasis by degrading dysfunctional organelles and proteins [4,17,25]. However, in cancer, autophagy can act as either a pro-survival mechanism or a pro-death pathway, depending on the cellular context and environmental conditions. In nutrient-poor or hypoxic environments, autophagy promotes cancer cell survival by providing essential nutrients and energy substrates. This adaptive response enables cancer cells to withstand metabolic stress and resist apoptosis induced by therapeutic agents [3,17,26]. Alternatively, autophagy can induce a form of non-apoptotic cell death known as autophagic cell death [26]. This process involves excessive or prolonged autophagy leading to cellular self-digestion and eventual cell demise, independent of apoptosis. The dual nature of autophagy in cancer underscores its complexity and context-dependent effects, influencing tumor progression, therapeutic responses, and overall cellular fate modulation [27,28].

Programmed necrosis, once considered a chaotic and unregulated form of cell death [29], has recently emerged as a regulated process with distinct mechanisms, including necroptosis, ferroptosis, and pyroptosis [3,30,31]. Necroptosis, mediated by receptor-interacting protein kinases, occurs when apoptosis is inhibited or compromised, leading to necrotic cell death with inflammatory consequences [32]. Ferroptosis, characterized by iron-dependent lipid peroxidation and membrane damage, represents a novel form of regulated cell death implicated in cancer progression and therapy resistance [33]. Pyroptosis, triggered by inflammasome activation and caspase-1 cleavage, results in inflammatory cell death and immune responses [5,30]. The regulation of programmed necrosis in cancer is complex and context-dependent, involving cross-talk with other cell death pathways and interactions with the tumor microenvironment [30,31,34].

The interplay between different forms of PCD — apoptosis, autophagy, and programmed necrosis — is intricate and multifaceted. Cross-talk between these pathways can either promote or inhibit cell death, depending on the cellular context and environmental conditions. For example, apoptosis and autophagy can synergize to eliminate cancer cells under certain conditions [25], whereas in other scenarios, autophagy may promote cancer cell survival and therapy resistance in the absence of apoptosis [24,27,28]. Additionally, programmed necrosis can serve as a backup mechanism for apoptosis when caspase activation is impaired [32], contributing to the resilience of cancer cells against cell death signals.

Although numerous mechanisms drive the initiation of PCD, cancer cells have evolved a diverse repertoire of strategies to evade these processes. Having established an understanding of the various modalities of cell death, our focus will now shift towards exploring how cancer cells modulate these PCD pathways. Specifically, we will delve into the mechanisms through which cancer cells resist apoptotic signals, with a particular emphasis on extrinsic apoptosis.

## Extrinsic mechanisms of apoptotic resistance in cancer

Cancer cells are under constant stress, facing oncogenic stress, genomic instability, cellular hypoxia, and extracellular apoptotic signals [3,5,16]. Typically, cells undergo PCD in response to stress, but cancer cells often evade this response by disabling apoptotic pathways, which is a hallmark of cancer [16]. They achieve this by down-regulating pro-apoptotic factors like caspases or up-regulating apoptosis inhibitors such as inhibitor of apoptosis proteins (IAPs) [25]. Additionally, cancer cells can desensitize themselves to extrinsic apoptotic signals by modulating death receptors [5,23]. In this context, we will explore two critical extrinsic mechanisms of immune-mediated cell death evasion by cancer cells: granule-mediated cytotoxicity and receptor-mediated cytotoxicity.

Granule-mediated cytotoxicity, employed by cytotoxic T lymphocytes (CTLs) and natural killer (NK) cells, releases cytotoxic molecules like perforin and granzyme B toward target cells, inducing intrinsic apoptosis [35–37]. Cancer cells counteract this process by degrading granzyme B or inhibiting cytotoxicity via hypoxia-induced autophagy [38,39]. Interestingly, alterations in mechanical properties on the cell membrane, such as lipid order, has been shown to impact perforin binding and cytotoxicity, with low-order lipids favored for perforin-mediated apoptosis [40,41]. For example, breast cancer cells resistant to lymphocyte cytotoxicity exhibit elevated lipid order, rendering them less susceptible to perforin-induced lysis [40,42]. Other mechanical features such as cell stiffness have been shown to influence susceptibility to perforin, with softer cancer cells evading T cell cytotoxicity [43].

Granule-mediated cytotoxicity serves as the primary mechanism for eliminating target cells in the presence of a large number of T cells. However, this process relies on CTLs to initiate target recognition, leading to the expansion of specific T cell populations capable of identifying target cells through specific peptides presented with the major histocompatibility complex class I (MHC-I) [44]. This presentation of peptides by MHC-I is indispensable for the binding of death receptors to ligands, initiating extrinsic apoptosis.

Cancer cells paradoxically express both the Fas (CD95) death receptor and Fas ligand (FasL), yet often exhibit resistance to Fas-mediated apoptosis [23,45,46]. Fas, a surface receptor from the tumor necrosis factor receptor (TNFR) superfamily, is primarily recognized for initiating cell death upon binding to its ligand, FasL. Despite abundant Fas expression on cancer cells, they often resist this apoptotic pathway, enabling them to evade cell death mechanisms [45,47]. Common evasion strategies involve the down-regulation of key components of the Fas signaling cascade, such as caspase-8 or Fas-associated death domain, which are essential for transmitting apoptotic signals initiated by Fas activation. Additionally, cancer cells often up-regulate cellular FLICE inhibitory protein, a potent inhibitor at the DISC, thereby preventing caspase-8 activation and subsequent apoptosis despite Fas receptor engagement [48]. Deregulation of B-cell lymphoma 2 (Bcl-2) family proteins or inhibitors of apoptotic proteins contributes to the loss of apoptosis signaling through Fas, promoting tumor survival [49]. Cancer cells also use FasL expression to indirectly target immune cells, inducing apoptosis in Fas-expressing CD8+ T cells and evading immune surveillance [50,51]. Remarkably, Fas activation can even boost cancer cell survival by enhancing their motility and invasiveness [45].

Similar to granule-mediated killing, receptor-mediated killing by CTLs or NK cells relies on the mechanical features of the cell membrane. The interaction between T-cell receptors (TCRs) of CD8+ T cells and MHC-I peptides is crucial for initiating apoptosis [44]. Soft membranes prevent effective TCR-MHC-I-peptide interactions, leading to insufficient downstream apoptotic signaling [52,53]. Moreover, MHC-I molecules are localized to higher order lipid regions, and depletion of cholesterol disrupts CTL recognition of MHC-I peptide complexes [36].

These findings suggest that cancer cells evade apoptosis not only through the regulation of biochemical players but also by modulating mechanical features, such as membrane stiffness. In the following section, we will examine the roles of receptor-mediated cytotoxicity, with a particular focus on how endocytosis contributes to cancer cell death. Finally, we will discuss strategies designed to enhance apoptotic sensitivity in these cells through targeting endocytosis.

## Roles of endocytosis in apoptotic evasion

Endocytosis plays a crucial role in various cellular processes, including nutrient uptake, receptor internalization, and signal transduction [54,55]. Defined as the process by which cells engulf extracellular molecules and particles by forming vesicles derived from the plasma membrane, endocytosis serves as a fundamental mechanism for maintaining cellular homeostasis and regulating cell signaling pathways [8,56,57].

Endocytosis is tightly regulated by various cellular factors, including membrane composition, cytoskeletal dynamics, and signaling pathways [56,58,59]. Membrane lipid composition, particularly the presence of cholesterol and sphingolipids, influences the formation and stability of endocytic vesicles [60]. Moreover, cytoskeletal elements such as actin filaments and microtubules provide the structural framework necessary for vesicle formation and intracellular trafficking [56,58]. Signaling molecules such as small GTPases, including dynamin and Rab proteins, regulate the budding and fusion of endocytic vesicles with target membranes [54].

There are several types of endocytosis, each serving distinct functions in cellular physiology [61]. CME is the most well-characterized form, involving the formation of clathrin-coated vesicles that transport cargo molecules into the cell [54,58,59]. Caveolae-mediated endocytosis occurs through invaginations of lipid raft domains enriched in caveolin proteins, facilitating the internalization of specific membrane components [62]. Additionally, macropinocytosis involves the nonspecific uptake of extracellular fluid and solutes through large, actin-driven membrane protrusions called macropinosomes [61].

CME, in particular, plays a significant role in oncogenesis and cancer cell proliferation [7,63]. Genetic mutations affecting endocytic proteins have been implicated in leukemia, underscoring the importance of endocytosis in cancer pathogenesis [64]. Moreover, posttranslational ubiquitination of endocytic proteins and receptors serves as a sorting signal in this pathway, influencing cellular processes crucial for cancer progression [64,65]. For example, ubiquitination regulates the internalization and trafficking of receptors such as epidermal growth factor (EGFR), impacting downstream signaling pathways that promote tumor growth and metastasis [66,67]. Additionally, ubiquitination of the E3 ubiquitin ligase Nedd4 can modulate the stability and function of CME machinery, affecting the turnover of membrane proteins involved in cancer cell signaling and survival, including EGFR [65,68]. Active Src kinase has been shown to promote degradation of Cbl, an important regulator of CME, resulting in elevated EGFR expression and signaling in tumors [66,67].

Tumor cells significantly diverge from normal cells in their cell membrane's structure and composition, resulting in the development of distinct signaling pathways that provide them with a survival edge [9]. Endocytosis plays a pivotal role in this process, as it can selectively engage in the uptake of extracellular molecules, thereby influencing apoptotic pathways and directly affecting cancer cell survival. By internalizing death receptors such as Fas/CD95 and TNFRs, cancer cells can sequester these receptors away from the cell surface, preventing their engagement with extracellular ligands and subsequent initiation of apoptotic signaling cascades [69,70]. Furthermore, endocytosis facilitates the internalization of anti-apoptotic proteins, such as Bcl-2 family members and IAPs, which inhibit pro-apoptotic signaling pathways and promote cell survival [25,71]. Endocytosis also influences cancer immunity by modulating the presentation of tumor-associated antigens. Down-regulation of surface display of MHC-I, facilitated by endocytosis, can impede T-cell-mediated cytotoxicity and promote immune evasion by tumors [72].

Concurrently, cancer cells evade immune surveillance by decreasing ‘eat me’ signals that promote their engulfment, such as exposure of phosphatidylserine on the outer membrane, modifying surface glycosylation patterns and epitopes of intercellular adhesion molecules, while increasing signals that inhibit phagocytosis (such as CD47, PD-L1, and beta-2 microglobulin) [73]. Alternatively, cancer cells release ‘find me’ signals that recruit monocyte or macrophage recruitment toward apoptotic cells, including lipid lysophosphatidylcholine, sphingosine 1-phosphate, fractalkine CX3CL1, and nucleotides ATP and UTP [73]. Although these signals can facilitate the efficient removal of these dying cells before they undergo secondary necrosis, which can trigger inflammation and tissue damage, cancer cells can evade detection and clearance due to their lack of expression of ‘eat me’ signals [73,74].

Dysregulating endocytic pathways has been linked to the altered expression and activity of key oncogenes and tumor suppressor genes, further influencing cancer cell fate [6]. In colon cancer, inhibiting CME has been found to impede tumor growth and enhance the therapeutic efficacy of immune checkpoint blockade, indicating that selective targeting of endocytic pathways could be a viable strategy in cancer treatment [72]. In addition, inhibiting endocytosis, particularly of death-inducing proteins [12], could enhance antitumor efficacy by preventing immune evasion mechanisms employed by cancer cells. Understanding the intricate mechanisms underlying endocytic regulation and its impact on apoptotic signaling pathways is essential for developing targeted therapeutic strategies aimed at overcoming cancer resistance to PCD.

## Inhibiting endocytosis sensitizes cancer cells to PCD

**Figure 1.**
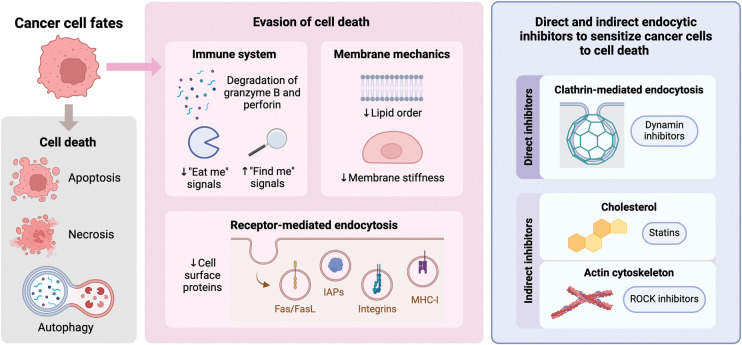
Schematic representation illustrating the potential therapeutic strategy of inhibiting endocytosis in cancer cells. Inhibiting endocytosis can block the internalization of death receptors, restore immune surveillance, and improve the delivery of therapeutics to sensitize cancer cells to programmed cell death. Figure created by Biorender.com.

Endocytic dynamics are increasingly recognized as a valuable target in anticancer strategies, mainly because of their role in facilitating targeted and efficient drug delivery [13] (Figure 1). Targeted drug delivery systems aim to minimize off-target effects, overcome multidrug resistance, ensure specific distribution to cancerous tissues, and improve the permeability of anticancer agents across cell membranes, ultimately enhancing the vulnerability of cancer cells to treatment [75]. Endocytosis plays a vital role in the uptake of drug-delivery vehicles, allowing therapeutics such as antibody-drug conjugates (ADCs) and radioligands to be efficiently transported into tumor cells [76].

One promising approach to target endocytosis in cancer is to inhibit the GTPase dynamin, a central regulator of multiple endocytic pathways [77]. In particular, dynamin-1, typically neuron-specific, has been shown to be activated in nonneuronal cells via cancer-relevant signaling pathways, establishing a feedback loop between CME and signaling to enhance cancer cell survival, migration, and proliferation [78,79]. Chemical inhibitors of dynamin, such as Dyngo [80], Dynasore [81], and phenothiazine [82] block the cell's GTPase activity, disrupting a wide range of dynamin-dependent endocytic processes. This inhibition of endocytosis has shown remarkable efficacy in suppressing proliferation and inducing apoptotic cell death across various cancer cell lines, including leukemia and lymphoma [83,84]. Notably, the disruption of endocytosis through dynamin inhibition has also been observed to overcome chemoresistance in leukemia stem cells, highlighting its potential to sensitize even the most recalcitrant tumor cells to cell death [85].

Beyond dynamin, other endocytic targets have been explored as a means to sensitize cancer cells. Compounds like Pitstop, which interfere with the clathrin-mediated endocytic machinery [86,87], have demonstrated the ability to enhance cancer cell susceptibility to cell death [88]. However, despite mutations in clathrin heavy chain that would theoretically block its supposed binding site, Pitstop 2 inhibits endocytosis indiscriminately [87,89–91]. This non-specific action indicates that Pitstop 2 may not be suitable for clinical use. Although Filipin III, which blocks caveolae/raft-mediated endocytosis [92], has been shown to overcome EGFR inhibitor resistance in lung cancer cells [93], it would not be effective on cells such as PC3 cells that lack cavin-1, a protein essential for caveolae formation [94]. These findings underscore the importance of various modes of endocytic regulation and combination therapies in modulating the response of cancer cells to targeted therapies.

The modulation of endocytosis can also be achieved through indirect approaches that alter the biophysical properties of the cell membrane [95–97]. Cancer cells often exhibit distinct membrane characteristics, such as altered cholesterol content and fluidity [98,99], which can significantly impact endocytic dynamics and the associated signaling cascades crucial for their survival. Agents such as statins, which target membrane cholesterol, have demonstrated anticancer effects in both preclinical and clinical studies. Lovastatin, simvastatin, and rosuvastatin, have shown promise in preclinical studies by temporarily enhancing tumor cell surface receptor density, thereby increasing the accumulation of monoclonal antibodies used in cancer therapies [94]. Lovastatin has also been reported to decrease markers associated with metastasis in breast cancer cells [100]. Furthermore, lovastatin has been shown to enhance apoptosis in brain cancer cells by increasing the activity of doublecortin, a brain-specific gene [101]. In some clinical studies, simvastatin, either used alone or in combination with other chemotherapeutic agents, has been demonstrated to significantly improve treatment outcomes and reduce mortality rates in patients with certain types of cancer [102–104].

Cancer cells are notably softer compared with healthy cells, facilitating rapid membrane remodeling during cancer progression [105–107]. This reduced stiffness is crucial to explain the observed increase in endocytosis in many cancer cells, particularly in a localized manner, which may be attributed to variations in local membrane composition, tension, and cooperative processes like actin remodeling [59]. These attributes play a vital role not only in facilitating cancer cell survival, invasion, and metastasis but also significantly impact the interactions between cancer cells and immune cells. For instance, T cells demonstrate diminished cytoskeletal forces and produce fewer effector cytokines when interacting with softer surfaces [53]. Thus, beyond biochemical immune checkpoints, mechanical checkpoints play a vital role in T cell-mediated cytotoxicity against cancer cells.

There has been growing interest in investigating Rho-associated kinases (ROCK) inhibitors as potential therapies for cancer. ROCK play a pivotal role in regulating the actomyosin cytoskeleton and contractile force generation [108]. This ROCK-driven contractility governs various cellular processes, including cell morphology, migration, invasion, proliferation, immune responses, and apoptosis resistance [109–111]. Inhibiting ROCK leads to increased membrane tension, which subsequently reduces endocytic dynamics [12]. Currently, several ROCK inhibitors such as Fasudil, Netarsudil, Belumosudil, and Ripasudil are approved for clinical use, primarily for treating hypertension [112,113]. While clinical trials using these inhibitors for cancer treatment have not yet been successful, numerous preclinical studies suggest that ROCK inhibition, when combined with chemotherapies, targeted therapies, and immunotherapies, leads to enhanced responses [113,114]. The promise of ROCK inhibitors lies in their ability to modulate the tumor microenvironment, improve drug delivery, and sensitize cancer cells to apoptosis, which preclinical models have shown to be effective in overcoming resistance mechanisms [113]. We have recently demonstrated that ROCK inhibition with Fasudil increases membrane tension in cancer cells and facilitates apoptosis by promoting the retention of Fas receptors on the cell surface [12]. This reduction in endocytosis has been observed to retain Fas receptors across multiple cancer cell lines without altering normal cells, enhancing sensitivity to the soluble Fas ligand and inducing cell death in two-dimensional culture, organoids, and *in vivo* models [12].

A word of caution is warranted when using endocytosis disruptors, whether genetic or pharmaceutical, to study endocytic regulators in cancer. Many small-molecule inhibitors lack specificity, disrupting multiple endocytic pathways [115]. Common strategies like altering membrane lipid composition or receptor distribution impact all endocytic pathways and essential signaling [94,116]. Inhibiting one pathway may up-regulate alternative routes, such as dynamin-independent endocytosis when dynamin is inhibited [117]. Broad dynamin targeting results in poor selectivity and off-target effects [118]. For instance, Dyngo has been shown to inhibit Trop2 endocytosis in prostate cancer cells, which can potentially reduce the effectiveness of Trop2-targeting ADCs [76,119]. Agents targeting membrane cholesterol like statins, while promising in cancer treatment, may interfere with uptake mechanisms and signaling due to altered fluidity, and affect cytoskeleton organization [120]. Methyl-β-cyclodextrin targeting cholesterol-rich lipid rafts is limited by cytotoxicity [121]. Manipulating intracellular cholesterol trafficking has shown efficacy in slowing melanoma growth, but strategies must carefully balance specificity and safety considerations [122].

Nevertheless, despite these challenges, targeting endocytosis to sensitize cancer cells to PCD remains a worthwhile endeavor in cancer therapy. Combining endocytic inhibitors with therapies such as ADCs and monoclonal antibodies holds promise for enhancing treatment efficacy while reducing off-target toxicity [85,94,123]. Temporary inhibition of CME can prevent the internalization of ADCs, increasing their retention on the cell surface, which in turn enhances antibody-dependent cellular cytotoxicity [90]. When this endocytosis inhibition is lifted, it has been shown that the ADC payload is then delivered to the endosomes in *ex vivo* tumor samples, enhancing its effectiveness while minimizing adverse effects on normal tissues [124]. By using endocytic inhibitors that offer transient and reversible inhibition, such as the dopamine receptor inhibitor prochloroperazine [82,123], systemic effects can be mitigated, ensuring the inhibition is cell-specific and temporary.

Disrupting dysregulated endocytic pathways that support tumor growth and survival holds promise for improving treatment outcomes across various cancer types. Endocytic inhibitors, whether administered alone, in combination with ADCs or radioligands, represent a critical strategy in cancer therapy. However, the journey to identifying optimal candidates for clinical use will require extensive research into their effects on the entire metastatic process. Addressing concerns of specificity, dosage, timing, safety, and the relevance of *in vivo* models is paramount to study and treat dysregulated endocytosis within the tumor microenvironment. As our understanding of the intricate relationship between endocytosis and cancer cell biology continues to evolve, the development of more selective and potent agents targeting these pathways holds the potential to significantly improve cancer treatment outcomes in the future.

## Perspectives

- Cancer cells evade PCD through various mechanisms, enabling unchecked survival and proliferation. Endocytosis plays a key role in regulating PCD pathways, providing opportunities to target this process and sensitize cancer cells to cytotoxic agents.
- Inhibiting endocytosis can prevent internalization of death receptors, restore immune surveillance, and enhance delivery of therapeutic payloads, rendering cancer cells more susceptible to PCD.
- Investigating the intricate relationship between endocytic trafficking and apoptotic signaling pathways is crucial for identifying new targets in cancer therapy. There is a growing demand to explore combined approaches that merge endocytic modulation with chemotherapies, targeted therapies, and immunotherapies to improve anticancer effectiveness.

## Abbreviations

ADC: antibody-drug conjugate
CME: clathrin-mediated endocytosis
CTL: cytotoxic T lymphocytes
DISC: death-inducing signaling complex
EGFR: epidermal growth factor
FasL: Fas ligand
IAP: inhibitor of apoptosis protein
MHC-I: major histocompatibility complex class I
NK: natural killer cells
PCD: programmed cell death
ROCK: Rho-associated kinases
TCR: T-cell receptors
TNFR: tumor necrosis factor receptor
